# Newly designed PCR assays based on the *ema-10* and *ema-11* genes confirm the circulation of *Theileria haneyi* in horses in Brazil

**DOI:** 10.1590/S1984-29612025049

**Published:** 2025-10-17

**Authors:** Rosangela Zacarias Machado, Marcos Rogério André, José Gomes Pereira, Maria do Socorro Costa Oliveira, Larissa Sarmento dos Santos Ribeiro, Carmen Zilda Pereira de Toledo, Luiz Ricardo Gonçalves

**Affiliations:** 1 Universidade Estadual Paulista – UNESP, Faculdade de Ciências Agrárias e Veterinárias – FCAV, Departamento de Patologia, Reprodução e Saúde Única, Vector-Borne Bioagents Laboratory – VBBL, Jaboticabal, SP, Brasil; 2 Universidade Estadual do Maranhão – UEMA, Departamento de Patologia, São Luís, MA, Brasil; 3 Universidade Estadual Paulista – UNESP, Faculdade de Ciências Agrárias e Veterinárias – FCAV, Jaboticabal, SP, Brasil; 4 Imunodot Diagnósticos Veterinários – IMUNODOT, Jaboticabal, SP, Brasil

**Keywords:** Babesia, equine piroplasmosis, equi merozoite antigen, Theileria, Babesia, piroplasmose equina, antígeno de superfície de merozoítos, Theileria

## Abstract

Equine Piroplasmosis (EP) is a tick-borne disease caused by the protozoan parasites *Babesia caballi*, *Theileria equi*, and *Theileria haneyi*, characterized by intravascular hemolysis and associated systemic illness. Although *T. equi* and *B. caballi* have been widely reported in some regions of Brazil, data from other states are limited. Additionally, despite reports of *T. equi* genotype C, currently recognized as *T. haneyi*, has been identified in Brazil, there are no investigations using *T. haneyi*-specific molecular tools. This study assessed the presence of these three agents in horses from Baixada Maranhense microregion (n = 34), northeastern Brazil, and in horses from an equestrian center (n = 12) in Guará, southeastern Brazil. Of 46 horse DNA samples, one (2.1%) from an imported animal in the equestrian center tested positive for *T. haneyi* in *ema-10* and *ema-11*-based PCR assays. Two animals tested positive for *T. equi* in a species-specific PCR (*ema*-*1*), and all samples were negative for *B. caballi*. BLASTn analysis showed *ema*-*10* and *ema-11* sequences shared 98.9% to 99.3% identity with *T. haneyi* detected in a horse at the U.S.-Mexico border. Despite the small sample size, this study confirms the presence of *T. haneyi* in Brazil and the need for monitoring imported animals.

## Resumo

A Piroplasmose Equina é uma doença transmitida por carrapatos, causada pelos protozoários *Babesia caballi*, *Theileria equi* e *Theileria haneyi*, caracterizada por hemólise intravascular e quadro clínico sistêmico. Embora *T. equi* e *B. caballi* sejam amplamente relatados em algumas regiões do Brasil, há informações limitadas em outros estados. Apesar de relatos do genótipo C de *T. equi*, atualmente reconhecido como *T. haneyi*, no Brasil, não há estudos com ferramentas moleculares específicas para *T. haneyi*. Este estudo verificou a presença desses três agentes em cavalos amostrados na microregião da Baixada Maranhense (n = 34), no nordeste, e de um centro equestre (n = 12) em Guará, no sudeste do Brasil. Dentre as 46 amostras analisadas, uma (2,1%) coletada de um animal importado, foi positiva para *T. haneyi* nos ensaios de PCR (*ema-10* e *ema-11*). Dois animais foram positivos para *T. equi* em PCR específico (*ema-1*), e todas as amostras foram negativas para *B. caballi*. A análise do BLASTn mostrou identidade de 98,9% a 99,3% com *T. haneyi* detectado em um cavalo amostrado na fronteira EUA-México. Embora o número de animais amostrados seja pequeno, o estudo confirma a presença de *T. haneyi* no Brasil e a necessidade de monitoramento dos animais importados.

## Introduction

Working equids, such as horses, donkeys, and mules, play an important role by supporting sectors including cattle raising, construction, tourism, mining, public transportation and other equine-assisted services, such as equine therapy. However, the health of these animals is often neglected (Valette, 2015).

Equine Piroplasmosis (EP) is a tick-borne disease caused by the hemoprotozoan parasites *Babesia caballi*, *Theileria equi*, and the newly discovered *Theileria haneyi* (Knowles et al., 2018). The EP affects both domestic and wild equids and the clinical appearance of the disease is characterized by intravascular hemolysis and associated systemic illness (Wise et al., 2014).

The infection caused by *T. equi* and *B. caballi* can lead to a similar clinical presentation yet *T. equi* and *B. caballi* are distinct in terms of virulence, life cycle, infection dynamics, drug resistance and persistence in horses (Zobba et al., 2008; Wise et al., 2014). On the other hand, *T. haneyi* demonstrated reduced clinical severity in spleen-intact or splenectomized horses (Knowles et al., 2018; Sears et al., 2022). In addition to infection dynamic studies, comparative genomic analysis of *T. equi* and *T. haneyi* has revealed that *T. haneyi* has undergone genomic reduction (Knowles et al., 2018).

Aside from the effects on the health of animals, *T. equi* infection has economic implications for international trade because infected horses are restricted from entering non-endemic countries (Coultous et al., 2023).

Despite of *T. equi* and *B. caballi* have been thoroughly reported in some areas of Brazil (Machado et al., 2012; Peckle et al., 2013; Braga et al., 2017; Vieira et al., 2018; Valente et al., 2019), limited information is available in some states. In addition, the knowledge on the occurrence and distribution of *T. haneyi* in Brazil is still incipient, particularly due the fact that previous studies did not use specific molecular screening for this piroplasmid. Although *T. equi* genotype C, currently recognized as *T. haneyi*, has been identified in Brazil (Peckle et al., 2018; Vitari et al., 2019), previous studies did not use *ema-10* and *ema-11*-based PCR assays, which should be more sensitive and specific to detect *T. haneyi*.

Therefore, the current study aimed to assess the presence of EP agents using molecular tools, including *T. haneyi*-specific PCR assays, in horses sampled in the microregion of Baixada Maranhense, northeastern Brazil, and in the city of Guará, southeast of Brazil.

## Material and Methods

### Animal blood sampling and DNA extraction

Blood samples were collected by convenience from 46 apparently healthy equids from two different sites in Brazil. Thirty-four blood samples were collected, by convenience, on EDTA-containing vials from horses in an endemic area for EP located in the microregion of Baixada Maranhense, in Maranhão State, northeastern Brazil. Similarly, 12 horses kept in an equestrian center located in the city of Guará, São Paulo state, southeast of Brazil, were also sampled during routine examinations. None of the sampled animals exhibited clinical signs suggestive of EP. DNA was extracted from 200 µL of each whole blood sample using the QIAamp DNA Blood Mini kit (QIAGEN, Valencia, California, USA), according to the manufacturer’s instructions.

### Molecular detection of piroplasmids and sequencing

Each horse blood DNA sample was used as a template in nested PCR assays (nPCR), using specific protocols based on the *ema-1* gene for *T. equi* and *rap1* gene for *B. caballi,* as previously described (Ikadai et al., 1999; Battsetseg et al., 2001; Nicolaiewsky et al., 2001). Briefly, 5 µL of DNA was used as a template in a 25 µL PCR reaction containing 2.5 µL of 10X PCR buffer, 1.5 mM MgCl_2_, 0.6 mM of a dNTP mix, 1.5 units of Taq DNA polymerase (Life Technologies), and 0.5 µM of each primer (Integrated DNA Technologies). *Theileria equi* and *B. caballi* DNA samples obtained from naturally infected horses (Braga et al., 2017) were used as positive controls.

For the detection of *T. haneyi,* primers specific to *T. haneyi* were designed through in silico analysis of *ema-10* (360 pb - MG652753), *ema-11* (580 bp - MG652754), and *ema-12* (850 pb - MG652755) sequences available in GenBank.

A semi-nested PCR (snPCR) - a variation of the nested PCR - was used as screening and performed with a final volume of 25 µL containing 2.5 µL of 10X PCR buffer, 1.5 mM MgCl_2_, 0.6 mM deoxynucleotide triphosphate (dNTPs) mixture, 1.5 U of Taq DNA polymerase (Life Technologies), five microliters of DNA template and 0.5 µM EMA11-F and EMA11-R primers in the first reaction (Table 1). The second reaction was carried out with a final volume of 25 µL containing 2.5 µL of 10X PCR buffer, 1.0 mM MgCl_2_, 0.6 mM deoxynucleotide triphosphate (dNTPs) mixture, 1.5 U of Taq DNA polymerase (Life Technologies), 1 μL of the amplified product in the first reaction and the primers EMA11-Fi and EMA11-R (0.5 µM) (Table 1).

**Table 1:** Description of primer sequences used in PCR assays for *Babesia caballi*, *Theileria equi*, and *Theileria haneyi*.

**Target**	**Primers (5’ – 3’)**	**Amplicon size (pb)**	**Anneling temperature**	**References**
**EMA-10**	EMA-10-F (CACTGGAGTCACCGTTGATG) EMA-10-R (GTGGCATCCTCAAGCTTCTC)	598	58 °C	Present study
	EMA-10-F (CACTGGAGTCACCGTTGATG) EMA-10-RI (GAACCAGACCTTCTCGTGGA)	360	60 °C	
**EMA-11**	EMA-11-F (TGGAGCACGTGAATGTTGAT) EMA-11-R (CCCATCATAGGCAACCTTGT)	647	55 °C	Present study
	EMA-11-Fi (TGCTGTTGAAAAGGTCGTTG) EMA-11-R (CCCATCATAGGCAACCTTGT)	580	56 °C	
**EMA-12**	EMA-12-F (TCTTTTTGGCCTGGTTTGTC) EMA-12-R (GCATTCTTCCTCCATGCTTC)	850	58 °C	Present study
**18S rRNA**	NBabesia1F (AAGCCATGCATGTCTAAGTATAAGCTTTT) 18SRev-TB (CCTCTGACAGTTAAATACGAATGCCC)	1500	60 °C	Bhoora et al. (2009)
	NBabesia1F (AAGCCATGCATGTCTAAGTATAAGCTTTT) BT18S3R (GAATAATTCACCGGATCACTCG)	800	58 °C	

A double-stranded DNA gBlock (IDT, USA) encoding a 647-bp *T. haneyi ema-11* gene fragment was designed for use as a positive control. snPCR amplifications were performed at 95 °C for 4 min followed by 35 repetitive cycles of 95 °C for 30 s, 55 °C for 30 s (second round: 56 °C for 30 s), and 72 °C for 30 s, followed by a final extension at 72 °C for 7 min. Samples positive for the above-described snPCR protocol were subsequently submitted to additional PCR assays targeting the *ema-10*, *ema-12* and *18S rRNA* (~1600 bp) genes. The primers and amplification conditions used in *ema-10*, *ema-12* and *18S rRNA*-based PCR assays are available in Table 1.

All PCR products that showed high intensity of bands with expected sizes were purified (Silica Bead DNA Gel Extraction kit - Thermo Scientific), submitted to sequence confirmation and the sequences analyzed by BLASTn.

## Results and Discussion

Equine piroplasmosis caused by hemoparasites of the *Babesia* and *Theileria* genera has important clinical and economic impacts worldwide. In the present study, despite the small number of horses sampled, we reported the occurrence of *T. equi* and *T. haneyi* in horses sampled in Brazil. In addition, our findings underscore the necessity and significance of maintaining and expanding animal health surveillance programs.

Out of 34 horse DNA blood samples obtained from animals from Baixada Maranhense, northeastern Brazil, two (5.8%) were positive for *T. equi* in a species-specific PCR. On the other hand, none of 12 horses kept in an equestrian center located in the city Guará was positive for *T. equi*. The molecular occurrence of *T. equi* found in the current study was lower than reported previously in Rio de Janeiro (81%) (Peckle et al., 2013), Paraíba (50.4%) (Ferreira et al., 2016), Rio Grande do Sul (18.9%) (Vieira et al., 2018), and Paraná (24%) (Valente et al., 2019). Different epidemiological factors associated with properties (management and sampling area), animal characteristics (age and sex) and diagnostic tools (PCR vs. qPCR) may influence the molecular occurrence of *T. equi* observed (Peckle et al., 2013; Campos et al., 2019). However, due to the limited number of animals analyzed, the low prevalence herein reported should be analyzed with caution.

*Theileria haneyi* was first detected in a horse on the US-Mexico border (Knowles et al., 2018) and has since been reported in some countries, including South Africa (Bhoora et al., 2020), Nigeria (Mshelia et al., 2020), Egypt (Elsawy et al., 2021) and Argentina (Benitez-Ibalo et al., 2025). Despite reports showing the occurrence of *T. equi* sequences closely related to *T. equi* genotype C in Brazil (Braga et al., 2017; Peckle et al., 2018; Vitari et al., 2019), the presence of *T. haneyi* has never been formally reported to date.

In the present study, we detected *T. haneyi* DNA in an imported horse from Texas, US, and kept in an equestrian center in Brazil. The animal was positive in the *ema-10* and *ema-11*-based PCR and the BLASTn analysis of the amplified sequences (PV276183 and PV276184) showed that the sequences shared identity ranging from 98.9% to 99.3% with *T. haneyi* (MG652753 - MG652754) detected in a horse restrained at the U.S. – Mexico border (Knowles et al., 2018). Although relatively short (360–580 bp), the sequences contained SNPs that accounted for the observed divergence among them. This animal, an adult male of the Paint Horse breed, arrived in Brazil in 2003 and remained in the central-western region of São Paulo state for approximately 15 years. After this, it was sent to an equestrian center in the city of Guará, state of SP, in 2018, where it died in 2024 under unknown circumstances.

Recently, studies have provided evidence that *T. equi* genotype C represents a novel species, *T. haneyi*, while several other cryptic *Theileria* species have been collectively classified under *T. equi* (Knowles et al., 2018; Nehra et al., 2024). Thus, as *T. equi* genotype C and closely related sequences have been previously reported in horses in Brazil, further investigation using *T. haneyi*-specific molecular tools, such as the ones employed herein, may clarify the occurrence and distribution of this newly discovered Piroplasmida species in the Brazilian territory.

Unfortunately, the positive sample in the PCR assays targeting the *ema-10* and *ema-11* genes tested negative in the *18S rRNA* and *ema-12*-based PCR, precluding a more accurate characterization of the *T. haneyi* found in the current study. This finding may be attributed to sequence variation in the *18S rRNA*, affecting primer hybridization with the target region and, consequently, impairing the amplification of certain *Theileria* genotypes.

All 46 horse DNA blood samples obtained from two different sites were negative for *B. caballi*. Although *B. caballi* has been reported in several regions of Brazil, the prevalence is relatively lower than that observed for *T. equi* (Machado et al., 2012; Vieira et al., 2018; Valente et al., 2019), except in limited cases (Braga et al., 2017).

## Conclusion

The current study revealed the presence of *T. haneyi* and *T. equi* in an underexplored region of Brazil. Additionally, we describe three species-specific molecular assays for the detection and characterization of *T. haneyi*. Although previous studies have suggested that *T. haneyi* has limited virulence, our finding reinforces the need for monitoring of animals entering Brazil and highlight the importance of maintaining animal health surveillance programs.

## Data Availability

All data generated or analyzed during this study are included in this published article. DNA sequences have been deposited in GenBank, and accession numbers are provided within the manuscript. Any additional data will be made available by the corresponding author upon reasonable request.

## References

[B001] Battsetseg B, Xuan X, Ikadai H, Bautista JLR, Byambaa B, Boldbaatar D (2001). Detection of *Babesia caballi* and *Babesia equi* in *Dermacentor nuttalli* adult ticks. Int J Parasitol.

[B002] Benitez-Ibalo AP, Debárbora VN, Mangold AJ, Nava S, Sebastian PS (2025). Molecular genotyping of *Theileria* spp. detected in horses from Corrientes City, Argentina. Vet Res Commun.

[B003] Bhoora RV, Collins NE, Schnittger L, Troskie C, Marumo R, Labuschagne K (2020). Molecular genotyping and epidemiology of equine piroplasmids in South Africa. Ticks Tick Borne Dis.

[B004] Bhoora RV, Franssen L, Oosthuizen MC, Guthrie AJ, Zweygarth E, Penzhorn BL (2009). Sequence heterogeneity in the 18S rRNA gene within *Theileria equi* and *Babesia caballi* from horses in South Africa. Vet Parasitol.

[B005] Braga MSCO, Costa FN, Gomes DRM, Xavier DR, André MR, Gonçalves LR (2017). Genetic diversity of piroplasmids species in equids from island of São Luís, northeastern Brazil. Rev Bras Parasitol Vet.

[B006] Campos JBV, André MR, Gonçalves LR, Freschi CR, Santos FM, de Oliveira CE (2019). Assessment of equine piroplasmids in the Nhecolândia sub-region of Brazilian Pantanal wetland using serological, parasitological, molecular, and hematological approaches. Ticks Tick Borne Dis.

[B007] Coultous RM, Sutton DGM, Boden LA (2023). A risk assessment of equine piroplasmosis entry, exposureand consequences in the UK. Equine Vet J.

[B008] Elsawy BSM, Nassar AM, Alzan HF, Bhoora RV, Ozubek S, Mahmoud MS (2021). Rapid detection of equine piroplasms using multiplex PCR and first genetic characterization of *Theileria haneyi* in Egypt. Pathogens.

[B009] Ferreira EP, Vidotto O, Almeida JC, Ribeiro LPS, Borges MV, Pequeno WHC (2016). Serological and molecular detection of *Theileria equi* in sport horses of northeastern Brazil. Comp Immunol Microbiol Infect Dis.

[B010] Ikadai H, Xuan X, Igarashi I, Tanaka S, Kanemaru T, Nagasawa H (1999). Cloning and expression of a 48-kilodalton *Babesia caballi* merozoite rhoptry protein and potential use of the recombinant antigen in an enzyme-linked immunosorbent assay. J Clin Microbiol.

[B011] Knowles DP, Kappmeyer LS, Haney D, Herndon DR, Fry LM, Munro JB (2018). Discovery of a novel species, *Theileria haneyi* n. sp., infective to equids, highlights exceptional genomic diversity within the genus *Theileria*: implications for apicomplexan parasite surveillance. Int J Parasitol.

[B012] Machado RZ, Toledo CZP, Teixeira MCA, André MR, Freschi CR, Sampaio PH (2012). Molecular and serological detection of *Theileria equi* and *Babesia caballi* in donkeys (*Equus asinus*) in Brazil. Vet Parasitol.

[B013] Mshelia PW, Kappmeyer L, Johnson WC, Kudi CA, Oluyinka OO, Balogun EO (2020). Molecular detection of *Theileria* species and *Babesia caballi* from horses in Nigeria. Parasitol Res.

[B014] Nehra AK, Kumari A, Moudgil AD, Vohra S (2024). Revisiting the genotypes of *Theileria equi* based on the V4 hypervariable region of the 18S rRNA gene. Front Vet Sci.

[B015] Nicolaiewsky TB, Richter MF, Lunge VR, Cunha CW, Delagostin O, Ikuta N (2001). Detection of *Babesia equi* (Laveran, 1901) by nested polymerase chain reaction. Vet Parasitol.

[B016] Peckle M, Pires MS, Silva CB, Costa RL, Vitari GLV, Senra MVX (2018). Molecular characterization of *Theileria equi* in horses from the state of Rio de Janeiro, Brazil. Ticks Tick Borne Dis.

[B017] Peckle M, Pires MS, Santos TM, Roier ECR, Silva CB, Vilela JAR (2013). Molecular epidemiology of *Theileria equi* in horses and their association with possible tick vectors in the state of Rio de Janeiro, Brazil. Parasitol Res.

[B018] Sears KP, Knowles DP, Fry LM (2022). Clinical progression of *Theileria haneyi* in splenectomized horses reveals decreased virulence compared to *Theileria equi.*. Pathogens.

[B019] Valente JDM, Mongruel ACB, Machado CAL, Chiyo L, Leandro AS, Britto AS (2019). Tick-borne pathogens in carthorses from Foz do Iguaçu City, Paraná State, southern Brazil: A tri-border area of Brazil, Paraguay and Argentina. Vet Parasitol.

[B020] Valette D (2015). Invisible workers: the economic contributions of working donkeys, horses and mules to livelihoods.

[B021] Vieira MIB, Costa MM, de Oliveira MT, Gonçalves LR, André MR, Machado RZ (2018). Serological detection and molecular characterization of piroplasmids in equids in Brazil. Acta Trop.

[B022] Vitari GLV, Costa RL, Abreu APM, Peckle M, Silva CB, Paulino PG (2019). Genetic diversity of *Theileria equi* from horses in different regions of Brazil Based on the 18S rRNA gene. J Parasitol.

[B023] Wise LN, Pelzel-McCluskey AM, Mealey RH, Knowles DP (2014). Equine Piroplasmosis. Vet Clin North Am Equine Pract.

[B024] Zobba R, Ardu M, Niccolini S, Chessa B, Manna L, Cocco R (2008). Clinical and laboratory findings in equine piroplasmosis. J Equine Vet Sci.

